# Controversies in the Management of Endometrial Carcinoma

**DOI:** 10.1155/2010/862908

**Published:** 2010-06-22

**Authors:** Ying Zhang, Jian Wang

**Affiliations:** Department of Obstetrics and Gynaecology, Xijing Hospital, Fourth Military Medical University, Xi'an 710033, China

## Abstract

Endometrial carcinoma is the most common type of female genital tract malignancy. Although endometrial carcinoma is a low grade curable malignancy, the condition of the disease can range from excellent prognosis with high curability to aggressive disease with poor outcome. During the last 10 years many researches have provided some new valuable data of optimal treatments for endometrial carcinoma. Progression in diagnostic imaging, radiation delivery systems, and systemic therapies potentially can improve outcomes while minimizing morbidity. Firstly, total hysterectomy and bilateral salphingo-oophorectomy is the primary operative procedure. Pelvic lymhadenectomy is performed in most centers on therapeutic and prognostic grounds and to individualize adjuvant treatment. Women with endometrial carcinoma can be readily segregated intraoperatively into “low-risk” and “high-risk” groups to better identify those women who will most likely benefit from thorough lymphadenectomy. Secondly, adjuvant therapies have been proposed for women with endometrial carcinoma postoperatively. Postoperative irradiation is used to reduce pelvic and vaginal recurrences in high risk cases. Chemotherapy is emerging as an important treatment modality in advanced endometrial carcinoma. Meanwhile the availability of new hormonal and biological agents presents new opportunities for therapy.

## 1. Introduction

Endometrial carcinoma is the most common type of female genital tract malignancy. It is estimated that 42,160 cases of endometrial carcinoma were diagnosed in the United States in 2008 and 7780 women would die from the disease [1]. Since the primary symptom is abnormal uterine bleeding in postmenopausal women, most patients would have a better chance of survival if diagnosed at an early stage of the disease. However, there still remain a lot of challenges in the clinical treatment of endometrial carcinoma. At the diagnostic stage, the condition of the disease can range from excellent prognosis with high curability to aggressive disease with poor outcome. In this paper, our goals are to discuss current challenges in the management of endometrial carcinoma and to provide an overview of the new approaches that would help overcome these challenges.

## 2. Pathological and Biologic Type

Pathological examination is the cornerstone in diagnosing endometrial carcinoma. There are different types of endometrial carcinomas, as shown in Table 1. The endometrioid tumors are further classified according to the degree of morphological differentiation. As defined by the International Federation of Gynecology and Obstetrics (FIGO), endometrioid carcinoma of grade 1 consists of well-formed glands, with no more than 5% solid nonsquamous areas (areas of squamous differentiation are not deemed to be solid tumor growth). Carcinomas of grade 2 consist of 6–50% and grade 3 consists of more than 50% solid nonsquamous areas. The tumor is upgraded from grade 1 to 2, or from grade 2 to 3 if striking cytological atypia is found [2]. 

It is considered that the different molecular biology of the different histological type is probably related to different behavior and prognosis. With more understanding about biologic behavior of endometrial carcinoma, we know that histological grading is far from enough to evaluate degrees of malignancy of endometrial carcinomas. Although about 80% of all endometrial carcinomas are of the endometrioid type, several subtypes or variants of endometrioid carcinoma provide more valuable information for guiding therapy. Most of all, special subtypes may be associated with higher death rate, for example, uterine papillary serous tumors and clear cell carcinoma. On the basis of their Pathological and biologic features, endometrial carcinomas are classified into 2 subtypes [2].

About 80% of all endometrial carcinomas are type I carcinoma (endometrioid type), arise from atypical complex hyperplasia, which seems to affect mainly pre- and perimenopausal women and presents with less myometrial invasion, lower grade disease. The type I tends to arise in the setting of prior estrogen stimulation because it is usually estrogen receptor positive and associated with hyperestrogenism [3, 4]. Other associated findings include late onset of menopause, nulliparity, diabetes mellitus, and hypertension. The patients with Type I endometrial carcinoma have a better prognosis since the lesion is limited to the uterus in 70% of the cases; the 5-year survival rate of these patients is more than 85%. 

In contrast, type II tends to occur in elderly postmenopausal women with high risk of relapse and metastatic disease, often with aggressive histologies such as serous or clear cell [3, 4]. Type II endometrial carcinomas appear to be unrelated to high estrogen levels. These tumors are not oestrogen driven and often develop in nonobese women. Type II endometrial carcinomas appear to be associated with endometrial atrophy; the histological type is either poorly differentiated endometrioid or nonendometrioid. A high proportion of tumors, even those with little or no myometrial invasion, have extensive extrauterine spread with complete surgical staging. More than 60% of patients with type II endometrial carcinoma present with advanced disease; 5-year survival is 43% for patients with stage III disease and 3% for patients with stage IV disease. Without adjuvant chemotherapy or vaginal brachytherapy, the recurrence rate is 23% in patients with stage I disease [4].

The molecular basis for different progression of these two subtypes is still unknown. However, a lot of clinical observations exhibited that gene alterations are specific for carcinomas of types I and II, which supports a dualistic model of endometrial carcinogenesis [5–10]. Type I endometrial carcinomas display a high incidence of alterations in KRAS oncogene, PTEN tumor suppressor gene [5, 6, 11–13], the *β*-catenin gene [14, 15], as well as defects in mismatch repair that results in microsatellite instability [10, 16]. In contrast, type II endometrial carcinomas are more likely to be characterized by p53 mutation and ERBB-2 (HER-2/neu) expression, and less commonly associated with E-cadherin and widespread aneuploidy [17–21]. However, there is some discrepancy in gene alterations report between two types of endometrial carcinomas (i.e., BUB1, CCNB2, MYC, STK15, etc.) [22]. Wong et al. performed an integrated, genome-wide analysis of gene expression in endometrioid adenocarcinomas and compared with normal endometrium controls. Supervised analysis identified 15 genes significantly upregulated and 132 genes downregulated in endometrial carcinoma, as compared with normal control. The gene expression profiles in endometrial carcinoma were classified in mutually dependent 6 function sets, resulting in 10 biological processes according to gene ontology. The gene ontology analysis showed that endometrial carcinogenesis underwent complete down-regulation of integrin binding and cell adhesion activity. Gene pathway analysis revealed the interaction among the genes of interest and its role in the endometrial carcinogenesis. The results from this preliminary study highlight novel molecular features of endometrioid endometrial carcinoma [23].

These data indicated that distinct patterns of gene expression characterize various histological types of endometrial carcinoma. An understanding of the molecular heterogeneity could potentially lead to better individualization of treatment in the future. Although some inconsistencies between single-gene and the whole-genomic approach have been observed, gene-array studies should be useful to disentangle molecular pathways and to identify potential targets for molecular-based treatments.

## 3. Diagnostic Approach

Endometrial carcinoma presents with abnormal uterine bleeding in 90% of patients. But other diseases could also cause abnormal uterine bleeding such as endometrial hyperplasia, and endometrial polyps. Proper treatment requires adequate preoperative work-up consisting of histopathology confirmation and imaging. The clinical approach to postmenopausal bleeding requires prompt and efficient evaluation to exclude or diagnose carcinoma. 

One of the most convenient methods of achieving this is transvaginal ultrasound. Transvaginal ultrasonography can be useful in the triage of patients in whom endometrial sampling was performed but tissue was insufficient for diagnosis. Endometrial thickness is the most valuable parameter to prognosticate both endometrial carcinoma and any endometrial pathology (sensitivities of 90% and 89%, and specificities of 79% and 94% with optimal cutoffs of 9.6 and 7.7 mm, resp.) [24]. The majority of these studies reported that a thin (4-5 mm) endometrial measurement on transvaginal sonography can exclude malignancy in the majority of postmenopausal women with vaginal bleeding. This has a negative predictive value of 96% when the endometrial echo is ≤4 mm thick, whereas an echo >4 mm indicates the need for a biopsy [25]. 

When scanning demonstrates the possibility of pathology, outpatient hysteroscopy and biopsy are the gold standard for investigating the endometrial cavity [26]. Hysteroscopy, a significantly more accurate diagnostic method for the detection of endometrial pathology than transvaginal ultrasonography (TVS), has better specificity and should be considered for all patients with abnormal uterine bleeding with an endometrial thickness of more than 4 mm. For women showing abnormal or suspicious lesions, it is necessary to perform hysteroscopy with eye-directed biopsy because some cases of endometrial carcinoma are unlikely recognized by ultrasonography with an endometrial thickness less than 4 mm, the possibility of missing is 0.8% [27, 28]. It can be stated that there is a high level of concordance between findings of hysteroscopic studies and the directed endometrial biopsy [29]. But it is a pity that hysteroscopy is not warranted as a first line investigation for postmenopausal bleeding [30].

When the diagnosis is confirmed histopathologically, imaging is recommended to identify stages of the disease radiologically prior to surgery. The accuracy/sensitivity/specificity of TVS, CT, and MRI in detecting deep myometrial invasion were 89%/90%/88%, respectively. The sensitivity and accuracy of MRI in detecting deep myometrial invasion were significantly higher than those of TVS and CT [25]. For diagnosis of deep myometrial infiltration, cervical invasion, or both, MRI sensitivity and specificity were 56% and 85%; 47% and 83%; and 67% and 77%, respectively. However MRI has limited value in identifying patients with endometrial carcinoma who are at risk of lymph node metastasis [31].

Positron emission tomography (PET) is a new imaging technology in detection of subclinical nodal disease. Several investigators have demonstrated the value of PET in screening endometrial carcinomas [32, 33]. Recently, Signorelli et al. reported that patient-based sensitivity, specificity, positive predictive value, negative predictive value, and accuracy of 18F-FDG PET/CT for detection of nodal disease were 77.8%, 100.0%, 100.0%, 93.1%, and 94.4%, respectively. Nodal lesion site-based sensitivity, specificity, positive predictive value, negative predictive value and accuracy of 18F-FDG PET/CT were 66.7%, 99.4%, 90.9%, 97.2%, and 96.8%, respectively. It seems that 18F-FDG PET/CT is an accurate method for the presurgical evaluation of pelvic nodes metastases. High negative predictive value may be useful in selecting patients who only may benefit from lymphadenectomy in order to minimize operative and surgical complications [34]. Kitajima and his colleagues compared the accuracy of integrated 18F-FDG PET/CT with intravenous contrast medium in detecting pelvic and paraaortic lymph node metastasis in patients of uterine carcinoma with surgical and histopathological findings used as the reference standard. They found that FDG-PET is only moderately sensitive in predicting lymph node metastasis preoperatively in patients with endometrial carcinoma [35]. Horowitz has similar conclusion about the sensitivity and specificity of FDG-PET for detecting pelvic and paraaortic lymph node metastasis in patients with uterine corpus carcinoma before surgical staging. The sensitivity and specificity of FDG-PET were 60% and 98%, respectively. A notable question is this imaging modality should not replace lymphadenectomy, but may be helpful for patients on whom lymphadenectomy cannot be, or was not, performed [36].

## 4. Treatment Overview

The cornerstone of curative for patients with endometrial carcinoma is surgical treatment, including complete hysterectomy, removal of remaining adnexal structures, and appropriate surgical staging in patients considered at risk for extrauterine disease. During the last 10 years interest in endometrial carcinoma has increased considerably and investigations into the following areas have increased our understanding of how we could reduce the risk of acquiring the disease and how we could best use the surgical and nonsurgical treatments available to us:

 optimal use of adjuvant radiotherapy;
 effect of hormonotherapy;
 role of chemotherapy;
 effectiveness of lymphadenectomy;
 genetic predisposition to the disease; and
 influence of less common histotypes.

## 5. Surgical Therapy

Treatment has remained relatively unchanged over the last 40 years relying principally on surgery to achieve cure. Survival is heavily dependent on surgical stage, which is determined by the classification system adopted by the FIGO in 1988. The foundation of primary treatment is hysterectomy, during which nodal assessment and surgical staging offer the opportunity for the most accurate assessment/detection of occult extrauterine malignancy in all women whose disease appears clinically confined to the uterus. Although these tenets are universally acceptable, the integration and implementation of these concepts when performing the “proper or appropriate” surgical procedure remain contested.

## 6. Surgical Staging

Surgical staging of endometrial carcinoma was first recommended 20 years ago by the FIGO. The development of surgical staging in the management of endometrial carcinoma has arisen over the last several decades with anticipated benefits including prognostic information, tailoring of adjuvant treatment, and a possible therapeutic effect. Twenty years later, the FIGO Committee introduced changes in the staging criteria [37]. Firstly, The FIGO Committee recognized the favorable prognosis for both the former Stage IA and IB patients and elected to merge these substages. Furthermore, the ambiguity of defining cervical invasion, based on the involvement of the cervical mucosa only, was recognized and the Committee merged the former Stage IIA with Stage I disease. Secondly, the Committee eliminated the isolated positive peritoneal cytology criterion from the new staging system presumably based on the uncertain prognostic importance of isolated positive peritoneal cytology. Thirdly, the Committee incorporated some tumor characteristics (such as positive peritoneal cytology, invasion of the adnexa or vagina, or uterine serosa) by subdividing Stage IIIC patients into 2 different risk categories based on the presence (IIIC2) or absence (IIIC1) of metastatic disease in the paraaortic area. 

Recently, Mariani et al. focused on the examination of paraaortic metastases relative to the inferior mesenteric artery (IMA), and found that 77% of patients with paraaortic node involvement had metastases above the IMA, whereas nodes in the ipsilateral paraaortic area below the IMA and ipsilateral common iliac basin were declared negative in 60% and 71%, respectively. In 25 patients with paraaortic node metastases which gonadal veins were excised, 28% patients had documented metastatic involvement of gonadal veins or surrounding soft tissue. These data indicates the need for systematic pelvic and paraaortic lymphadenectomy up to the renal vessels including consideration of excision of the gonadal veins [38].

## 7. Lymphadenectomy

As will be referred to shortly, there has been a vigorous debate about the benefits of pelvic (plus or minus paraaortic) lymphadenectomy. Although the assessment of the pelvic and paraaortic lymph nodes has been recommended since 1988, FIGO failed to define either the anatomical extent of the lymphadenectomy or the number of lymph nodes harvested to be considered adequate for the assessment of pelvic and paraaortic node basins. This question is further complicated when people try to assess the adequacy of lymphadenectomy that was performed.

There is also lack of consensus on the extent of surgical staging in endometrial carcinoma. Some authors suggest performing complete pelvic and paraaortic lymphadenectomy on all endometrial carcinoma patients because positive lymph nodes (including isolated paraaortic lymph nodes) are common in all grades [39]. It is reported that the carcinoma related survival and the recurrence free survival were better with standard surgery plus lymphadenectomy than with with adjuvant radiotherapy in treating the endometrioid adenocarcinoma type at high risk [40]. Other studies have assessed readily discernible parameters intraoperatively to identify patients having an extremely low probability of lymphatic spread in order to minimize under- and over-treatment [38, 41]. A recent report by Mariani et al. showed that sixty-three (22%) of 281 patients undergoing lymphadenectomy had lymph node metastases: both pelvic and paraaortic in 51%, only pelvic in 33%, and isolated to the paraaortic area in 16%. Furthermore, 77% of patients with paraaortic node involvement had metastases above the inferior mesenteric artery. Conversely, lymphadenectomy does not benefit patients with grade 1 and 2 endometrioid lesions with myometrial invasion ≤50% and primary tumor diameter ≤2 cm [38]. In the most recently published prospective randomized trials that aimed to test the therapeutic benefit of lymphadenectomy, Benedetti Panici reported that both early and late postoperative complications occurred more frequently in patients who had received pelvic systematic lymphadenectomy. Although systematic pelvic lymphadenectomy statistically significantly improved surgical staging, it did not increase disease-free or overall survival rate [42].

Researchers are concerned about the results of ASTEC surgical trial that showed no evidence of benefit in terms of overall or recurrence-free survival for pelvic lymphadenectomy in women of early endometrial carcinoma [43]. However, Amant et al. argued that there are several reasons why the ASTEC trial did not show improved overall survival with routine lymphadenectomy [44]. First, the number of lymph nodes resected was insufficient in many patients. Second, the high rate of inclusion of low-risk patients and the low number of lymph nodes removed are the reasons for the low rate of involved lymph nodes seen in the lymphadenectomy group. Third, the study group did not assess the paraaortic nodes. Fourth, the ASTEC trial was too small to detect an overall survival difference because the expected proportion of isolated pelvic lymph-node recurrences is as low as 2-3% in early endometrial carcinoma. 

Without clear standard recommendations, surgical staging will continue to be a confusing topic, with no appropriate quality control. There are still many unanswered questions. Are there a critical minimum number of nodes that should be resected? Do the paraaortic nodes always need to be resected? Should the histologic type of uterine carcinoma determine the extent of lymphadenectomy? Do the modern robotic-assisted or laparoscopic approaches provide surgeons adequate exposure to perform sufficient lymphadenectomy? The ideal surgical staging for endometrial carcinoma remains a subject of active debate. We are hoping that more prospective randomized trials will solve them.

## 8. Laparoscopy

The standard surgical surgery of endometrial carcinomas includes total hysterectomy, bilateral salpingo-oophorectomy, and lymphoidectomy. However, the application of laparoscopy in the management of gynecologic malignancy has received much attention and given rise to considerable debate. During the past few years, several investigators have demonstrated that total or vaginally-assisted laparoscopic hysterectomy, associated with laparoscopic pelvic lymphadenectomy, represents a valid alternative to open surgery [45–48]. The potential health gain of performing a laparoscopic hysterectomy instead of an abdominal hysterectomy in patients with early stage endometrial carcinoma is expected in lower rate of intraoperative complications, less blood loss; lower transfusion rate and haemoglobin decrease, shorter hospital stay, as well as a faster return of bowel activity and quicker return to activities in daily life. Nevertheless, laparoscopic hysterectomy does not seem to modify the disease-free survival and the overall survival, laparoscopic approach is an effective procedure for treating early stage endometrial carcinoma [49–51]. Randomized trials and long-term follow-up at various medical centers are necessary to evaluate the overall oncologic outcomes of this procedure.

## 9. Radiotherapy

Due to the difficulty in detecting cervical involvement preoperatively, treatment paradigms for stage II endometrial carcinoma often call for adjuvant radiotherapy postoperatively [52]. The debate regarding whether postoperative radiotherapy could improve survival has been fueled by multiple retrospective studies which have presented conflicting conclusions. 

Several studies suggested that survival rate increases if a surgery is performed in conjunction with adjuvant pelvic radiotherapy, external beam radiotherapy (EBRT) or brachytherapy (BT). For high-risk disease, the standard care has always been pelvic radiotherapy. Clearly, there are advantages as shown in meta-analyses and by the Cochrane group [53, 54]. In the Gynecologic Oncology Group's prospective evaluation of adjuvant radiation, which included patients with occult stage II tumors, radiation decreased the risk of pelvic recurrence [55]. In a report of 162 stage II endometrial carcinoma patients, Cohn et al. noted that the 5-year disease-free survival was improved (94% versus 76%) in patients who underwent radical hysterectomy [52]. Likewise, the studies by Rossi teams came to similar conclusions. They found that women with Stage IIIC endometrial carcinoma receiving adjuvant EBRT and EBRT/BT had improved overall survival compared with patients receiving no additional radiotherapy. When direct extension of the primary tumor was present, the addition of BT to EBRT was even more beneficial [56]. Up to date, Wright and his colleagues examined 1577 women with stage II endometrial adenocarcinoma and analyzed the role of radical hysterectomy and radiation in management of endometrial adenocarcinoma. They found that women who did not receive radiation were 48% more likely to die from their tumors. The benefit of adjuvant radiation is most pronounced in women with high-risk pathologic features who underwent radical hysterectomy [57]. 

In contrast, other investigators have been unable to show a survival benefit based on the type of surgical procedure performed. In the paper by Kong, there is undoubtedly a benefit in local control when adjuvant pelvic radiotherapy is given but again no survival advantage. This is further supported by a presentation at ECCO 2007 from Cornes and Johnson in which they showed that there is up to a 10% survival advantage for patients with IC G3 tumors treated with pelvic radiotherapy [58]. They have also shown that for low-risk patients adjuvant EBRT is probably detrimental whilst for intermediate-risk patients although there may be a small benefit for some patients, this is offset by additional morbidity leading to an overall neutral effect. There are also two papers looking at data from the Survival, Epidemiology, and End Results (SEER) database [59, 60]. Both Lee et al. and Chan et al. analyzed the SEER data and showed that patients with high-grade IC G3 tumors appeared to benefit but failed to show any benefit to other patients. The data from a prospective, multicenter randomized trial of 645 evaluable low-risk endometrial carcinoma patients was showed that the impact of postoperative brachytherapy on even the locoregional recurrence rate seems to be limited in patients with low-risk endometrial carcinoma. The overall recurrence rate and survival were similar in postoperative vaginal irradiation and surgery alone groups [61]. 

The fresh data of ASTEC/EN.5 randomized trials was published recently. There was no evidence that overall survival with external beam radiotherapy was better than observation. Combined data from ASTEC and EN.5 in a meta-analysis of trials confirmed that there was no benefit in terms of overall survival (hazard ratio 1.04; 95% CI 0.84–1.29) and can reliably exclude an absolute benefit of external beam radiotherapy at 5 years of more than 3%. Interpretation adjuvant external beam radiotherapy cannot be recommended as part of routine treatment for women with intermediate-risk or high-risk early-stage endometrial carcinoma with the aim of improving survival [62]. Meanwhile, we should notice that adjuvant external beam radiotherapy did result in a small reduction in isolated local recurrence, but this analysis only included women who had local recurrence alone, ignoring 65% of women who had local and distant recurrence at the same time, or distant recurrence alone. The small reduction in isolated local recurrence does not translate into an effect on overall or recurrence-free survival. 

Up to this point, it was emerging that patients with low-risk disease do not need any adjuvant treatment and can be treated by surgery and careful follow up. Patients with intermediate-risk disease are more problematic and may still be treated with external beam radiotherapy. Although the majority of retrospective data has not demonstrated a benefit for radiation, it has been suggested that women who undergo simple hysterectomy and are found with cervical disease may benefit from radiotherapy [63]. Feltmate et al. reported excellent outcomes in a series of 65 patients with stage II endometrial carcinoma, the majority of whom were treated surgically and followed by adjuvant radiation. In their cohort, 5-year disease-specific survival was 93% with recurrences in 15% [64]. Among 203 subjects with endometrial carcinoma, Sartori et al. noted a statistically significant improvement in 5-year survival from 74% with simple hysterectomy to 94% with a radical procedure [65].

Some clinical trials investigated the optimal of radiotherapy mode. It is considered that brachytherapy is a more convenient treatment than external beam radiotherapy and might be associated with less toxicity. In the PORTEC1-trial, the 5-year risk of vaginal and pelvic recurrence for high- intermediate risk patients was 19% without further treatment, compared to 5% after EBRT. Since most recurrences were located in the upper vagina, Phase II trials suggested vaginal brachytherapy (VBT) to be as effective as EBRT. PORTEC-2 is the first randomized trial comparing the efficacy of VBT and EBRT to determine which treatment provides optimal local control with best quality of life. The data suggested that vaginal brachytherapy is effective in preventing vaginal recurrence. Despite the slightly but significantly increased pelvic failure rate in the VBT arm, rates of distant metastases, OS, and RFS were similar. As indicated by the patient survey on quality of life after treatment, VBT was shown to be better than EBRT, VBT should be the treatment of choice for patients with high-intermediate risk endometrial carcinoma [66]. First results of the randomized PORTEC-2 trial are evaluation about quality-of-life (QOL) after pelvic radiotherapy or vaginal brachytherapy for endometrial carcinoma. Patients in the VBT group reported better social functioning (*P* < .002) and lower symptom scores for diarrhea, fecal leakage, the need to stay close to the toilet, and limitation in daily activities because of bowel symptoms (*P* < .001). Vaginal brachytherapy provides a better quality of life than external-beam radiotherapy for Endometrial Carcinoma, and should be the preferred treatment from a quality of life perspective [67]. 

Nevertheless, the data is important and add to our understanding of the optimal management of endometrial carcinoma. Data from these data banks and the Cochrane reviews may help to address the question of which is the optimal treatment for this group. A further approach is to withhold radiation in the intermediate-risk group and offer careful surveillance and use salvage radiotherapy for relapses confined to vagina or vault. In the meantime we should consider that either immediate external beam radiotherapy or a watch and see policy with salvage radiation should be the standard approach.

## 10. Chemotherapy

Chemotherapy is emerging as an important treatment modality in advanced endometrial carcinoma. The use of neoadjuvant chemotherapy resulted in a high rate (80%) of optimal interval debulking surgery for the treatment of endometrial carcinoma with transperitoneal spread [68]. GOG 122 was the first randomized study to demonstrate a survival advantage with chemotherapy in advanced stage endometrial carcinoma [69]. At 60 months, 50% of patients received doxorubicin and cisplatin chemotherapy were predicted to be alive and disease-free when adjusting for stage compared with 38% of patients who had whole-abdominal irradiation. The data from GOG 122 showed that combination chemotherapy had a survival advantage over whole abdominal radiotherapy in Stage III and IV endometrial carcinoma.

There are several studies focused on the toxicity, tolerability, and feasibility of delivering combination chemotherapy with subsequent radiation therapy to women with advanced endometrial carcinoma, and evaluate the long-term bowel toxicity. It is notable that GOG 122 study had an extremely high toxicity rate from chemotherapy (68% Grade 4 hematologic toxicity), including 8 treatment-related deaths. It is apparent that cisplatin and/or doxorubicin-based regimens are associated with unfavorable rates of toxicity [69]. A Phase I GOG study by Soper et al. indicates that treatments comprised of whole abdomenopelvic radiation with concomitant weekly cisplatin, followed by doxorubicin and cisplatin chemotherapy, had prohibitive toxicity and did not undergo further evaluation [70]. Bruzzone et al. reported a series of 45 women who received cisplatin and cyclophosphamide followed by radiotherapy, in which 10 women (22%) completed 3 cycles or less [71]. Duska et al. reported a pilot study for advanced stage disease, which included 3 cycles of paclitaxel, doxorubicin, and carboplatin, followed by radiotherapy [72]. All patients required G-CSF support, but 50% still experienced Grade 3 or 4 acute toxicity. In RTOG 9708, in which 4 cycles of cisplatin and paclitaxel were administered after completion of radiotherapy, acute Grade 3/4 toxicity was greater than 80% [73]. In comparison, Lupe et al. used the combined modality protocol comprised of carboplatin and paclitaxel with involved field radiotherapy had a much lower acute toxicity rate, and the compliance rate was very high [74].

Meanwhile, the use of chemotherapy alone has been associated with high rates of pelvic relapse, ranging from 18% to 47% [69, 75]. Recently, Takeshima et al. reported with postoperative adjuvant chemotherapy, recurrences occurred predominantly at distant sites in the absence of pelvic radiation in surgically staged grade 3 endometrial carcinoma. Estimated 5-year disease-free survival rates were 89.8% for patients with surgical stage I-II disease, 78.6% for those with surgical stage III disease, and 87.3% overall [76]. There is an emerging consensus that chemotherapy may be insufficient for reducing the risk of pelvic relapse although it appears to be an important component of treatment. Sovak et al. reported a pelvic relapse rate of 44% in patients with Stage III and IV disease who received 6 cycles of adjuvant carboplatin and paclitaxel, of whom only 5 (10%) also received adjuvant pelvic radiotherapy [77]. In RTOG 9708, the pelvic relapse rate was only 2%. It suggested that the addition of radiation to chemotherapy does appear to be associated with a lower rate of pelvic relapse [73]. However, in that study, 23% had Stage I and 16% had Stage II disease. The low rate of pelvic relapse may be partly attributed to the more favorable stage distribution. Alvarez Secord et al. published a large retrospective study of 356 Stage III and IV patients treated with radiation alone (48%), chemotherapy alone (29%), and combined modality therapy (23%) [78]. After adjusting for stage, age, grade, and debulking status, the hazard ratios (HR) for overall survival were 1.6 (95% CI 0.88–2.89) and 2.0 (95% CI 1.17–3.48) for chemotherapy and radiation alone, respectively, compared to combined modality therapy. Matsuura et al. reported most recently that combined treatment with radiotherapy/chemotherapy was associated with a better survival rate than chemotherapy alone (78% versus 62%, resp.). In Stage IIIc endometrial carcinoma, the combined use of radiotherapy and chemotherapy could reduce pelvic recurrence (33.3% and 7.1%, resp.) and was associated with a better survival rate than chemotherapy alone (78% versus 62%, resp.) [79]. Based on this concurrent carboplatin/paclitaxel and intravaginal radiation in surgical stage I-II serous endometrial carcinoma study, surgical staging followed by involved-field radiotherapy and carboplatin/paclitaxel is well tolerated and effective in stage I-II serous endometrial carcinoma [80]. Confirmation of these results on a larger number of patients with longer follow-up is still needed.

What is the optimized chemotherapy regimen is still a subject of debate. Historically, the treatments used have been a combination of a platinum and anthracycline, usually cisplatin and doxorubicin (AP), but this can be quite a toxic regime and is often poorly tolerated, therefore it is not ideal for combining with radiation therapy. Adding paclitaxel (TAP) usually needs growth factors to support the administration. Hellenic Co-operative Oncology Group (HeCOG) studied the drug regimen comprised paclitaxel, topotecan, and carboplatin in metastatic endometrial carcinoma. With G-CSF support, the drug regimen appears active with acceptable toxicity in patients with metastatic or recurrent carcinoma of the endometrium [81]. In relapsed disease, the GOG are currently evaluating TAP versus TC [82]. In addition, the optimal regimen remains to be defined as all of them (doxorubicin/cisplatin-AP, cyclophosphamide/doxorubicin/cisplatin-CAP, paclitaxel/carboplatin-TC, and paclitaxel/doxorubicin/ cisplatin-TAP) cause significant toxicity. Although randomized evidence is limited, the combination of carboplatin and paclitaxel has been commonly used in advanced endometrial carcinoma because of its manageable toxicity and excellent response rates (64–78%) [77, 83–88]. McMeekin et al. studied the maximum tolerated dose and feasibility of weekly cisplatin and paclitaxel chemotherapy administered concurrently with whole abdominal radiation therapy in women with high-risk endometrial carcinoma. A regimen of cisplatin 25 mg/m^2^ and paclitaxel 20 mg/m^2^ weekly with whole abdominal radiation therapy was determined to be feasible, but is associated with moderate acute and chronic gastrointestinal toxicity [89].

Further investigations are required to define the subgroup of patients who benefit from postoperative adjuvant chemotherapy. Two randomized clinical trials are in progress in order to obtain available evidence which can help clinicians make wise decisions on treatment options, such as adjuvant chemotherapy of patients with high-risk stage I and II, as well as stage IIIA endometrial carcinoma. GOG 209 is an ongoing study randomizing women with Stage III or IV endometrial carcinoma to either doxorubicin, cisplatin, paclitaxelwith G-CSF, or carboplatin and paclitaxel. Additionally, PORTEC 3 is an ongoing randomized Phase III trial comparing concurrent chemoradiation and adjuvant chemotherapy (4 cycles of carboplatin and paclitaxel) versus pelvic radiation alone in high risk and advanced stage disease. This study is timely and necessary to determine whether radiotherapy or chemotherapy improves overall survival and failure-free survival, compare the rates of severe (grades 3 and 4) treatment-related toxicity, pelvic and distant recurrence, and evaluate quality of life of patients with high-risk and advanced stage endometrial carcinoma.

## 11. Functional Preservation

Although the median age of patients with endometrial carcinoma is in the early 60s, approximately 5% of patients are younger than age 40 when diagnosed. In the presence of early staged endometrial carcinoma, most have favorable outcomes, thus their quality of life after treatment is as important a consideration as a cure of carcinoma. This issue is especially imperative when endometrial carcinoma is encountered in younger or reproductive ages when the afflicted woman has not achieved her fertility function. Despite being a critical issue, there are only a few studies with definite treatment guidelines or any evidence-based recommendations concerning conservative treatment for endometrial carcinoma.

Since early 1980s, there have been several reports on conservative treatment with progestins for early-stage endometrial carcinoma in young women. Most of them were small series and retrospective studies from single institutions. Response rates and recurrence rates varied (i.e., the response rate for endometrial carcinoma and atypical endometrial hyperplasia ranged from 57% to 76% and from 83% to 92%, respectively, and the recurrence rate ranged from 11% to 50%) [90–96]. Such variations were probably due to the differences in drugs used, dosage, and duration of treatment. Daily doses of megestrol acetate ranged between 10 and 400 mg, and that of medroxyprogesterone acetate (MPA) ranged between 200 mg and 800 mg. Nevertheless, there have been no prospective trials to investigate the optimal dosage, duration of treatment, curative rate of MPA treatment, or pregnancy rate after this therapy in young women with endometrial carcinoma and atypical endometrial hyperplasia. Therefore, Ushijima et al. conducted a multicenter, prospective phase II study on MPA treatment. Their prospective study conducted to clarify the accurate complete response (CR) rate of treatment with MPA at a fixed dose of 600 mg/d for 26 weeks, has demonstrated that the CR rate for endometrial carcinoma and atypical endometrial hyperplasia was 55% and 82%, respectively, and the recurrence rate was 57% and 38%, respectively [97]. In another prospective multicentric prospective study, Ushijima et al. evaluated the efficacy of fertility-sparing treatment by MPA for endometrial carcinoma and atypical endometrial hyperplasia. Complete response was found in 44% in endometrial carcinoma and 82% in atypical endometrial hyperplasia. 9 pregnancies and 4 normal deliveries have been recorded after MPA therapy. Twelve recurrences were found in 30 complete response patients (40%) between 7 to 22 months. Data showed that even in the complete response patients, close follow-up is required because of their high recurrence rate [98]. Recently, Signorelli et al. conducted a prospective study of conservative treatment in 21 young nulliparous women with grade G1 endometrial carcinoma stage IA or atypical complex hyperplasia. All were treated with a low-dose cyclic natural progestin therapy (200 mg/day from day 14–25) and encouraged to attempt pregnancy immediately. Overall response rate to progestin therapy was 57%. Nine women conceived spontaneously (43%) and 8 women with persistent disease or partial response to hormonal treatment. Three additional complete responses were observed after delivery [99].

A largely unanswered question is the safety of ovarian preservation in young women with endometrial carcinoma. First, estrogen production from the ovaries may stimulate microscopic foci of residual endometrial carcinoma. Although vitro data [100] has suggested that estrogen stimulates the growth of endometrial carcinoma cells and upregulates the expression of estrogen receptors, this concern has not been observed clinically so far. Several reports examined the use of estrogen replacement therapy in postmenopausal women with endometrial carcinoma. Yet, these studies have not demonstrated any increase in the risk of recurrence or death in women receiving estrogen replacement [101–103]. The new data published by Korean Gynecologic Oncology Group (KGOG) in 2009 suggest that ovarian preservation does not adversely impact the recurrence of early stage endometrial carcinoma [103]. The most influential report was a prospective trial of estrogen replacement therapy in more than 1,200 women with endometrial carcinoma conducted by the Gynecologic Oncology Group. Although the prospective trial was closed early, the absolute recurrence rate was only 2.1% (HR 1.27; 95% CI, and 0.92 to 1.77) [101]. The findings from these studies, as well as the data from Wright group, suggest that the risk of estrogenic stimulation of residual endometrial carcinoma is quite low, particularly in women with early-stage, low-risk lesions. The second potential risk of ovarian conservation is the presence of a coexisting synchronous primary tumor within the ovaries. Synchronous primary tumors of the endometrium and ovary are reported in approximately 5% of women with endometrial carcinoma [104]. However, among young women with endometrial carcinoma, the incidence of coexisting ovarian tumors is increased and has been reported with a range from 5% to 29% [104–107]. 

Although many studies have examined the risk of ovarian metastases in young women with endometrial carcinoma, there are no data to describe the safety of ovarian conservation. In 2009, Wright firstly reported that ovarian preservation is safe in young women with early-stage, low-grade endometrial carcinoma [108]. Their findings are notable in that ovarian preservation in premenopausal women with early-stage, low-grade endometrial carcinoma may be safe and not associated with an increased risk of carcinoma related mortality. Although the survival estimates suggest that ovarian conservation does not negatively impact outcome, it should be recognized that ovarian preservation may be associated with a two-fold or greater increase in mortality. Given the potential consequences of surgical menopause, further research to examine the safety of ovarian conservation for young women with early-stage endometrial carcinoma is clearly warranted. At present, the long-term risks and benefits of ovarian preservation should be carefully discussed with young women with endometrial carcinoma before hysterectomy.

## 12. Fertility Sparing

Although there is no known fertility-sparing surgical option for women with endometrial carcinoma, selected young patients of childbearing age with apparent early endometrial carcinoma who wish to preserve fertility may consider treatment with progestin therapy rather than surgery. If such treatment is contemplated, it is recommended that a thorough hysteroscopy and curettage be performed to rule out a worse lesion prior to initiation. A review of the literature indicates 101 patients with a median age of 29 years who were treated with progestin therapy rather than definitive surgery subsequently had 56 children [91]. Additionally, Gershenson et al. provided indirect evidence to support the recent concept of using the fertility-sparing or conservative surgery or therapy for malignancies in women that the use of conservative modalities can be applied in the management of endometrial carcinomas because there are many reports showing that endometrial carcinomas can be treated with a simple diagnostic dilatation and curettage followed by some potent hormone therapy, including a progestin agent, in highly selected young women who would like to preserve their fertility potential [109].

Recently, there have been a number of reports of women with uterine endometrial carcinoma who became pregnant and gave birth after the administration of medroxyprogesterone acetate (MPA) [93, 110–114]. Meanwhile subsequently assisted reproductive techniques such as transfer of embryos with intracytoplasmic sperm injection (ICSI) and preimplantation genetic diagnosis (PGD) may be valuable to achieve immediate pregnancy [115–117]. 

Gadducci et al. reviewed the related literature and confirmed that approximately three fourths of the women achieve a histologically documented complete response, with a mean response time of 12 weeks, but about one third of these subsequently developed a recurrence after a mean time of 20 months. Following high dose progestin therapy and confirmation of the regression of carcinoma, the patient might attempt to conceive spontaneously. However, assisted reproduction techniques might increase the likelihood of pregnancy and decrease the time interval to conception. Several successful pregnancies have occurred after a fertility-sparing treatment of endometrial atypical hyperplasia or endometrial carcinoma, more frequently with assisted reproductive technologies. The implementation of in vitro fertilisation techniques not only increases the chance of conception, but it may also decrease the interval to conception [118]. However, despite the achievements of these studies on fertility-sparing treatments, there are no definite treatment guidelines or any evidence-based recommendations and many questions still remain unanswered regarding the selection of patients. Nevertheless, the optimal dose or duration and curative rate of MPA therapy in endometrial carcinoma and atypical endometrial hyperplasia in young women are still uncertain. 

It is vital to choose appropriate patients with endometrial carcinoma to adopt ovarian preservation and fertility-sparing treatment. The best candidates for progestin therapy are women who have a relative hyperestrogenic state, which is thought to cause the malignancy. Indeed, some patients would not chose fertility-sparing treatment given the lack of data on oncologic safety. Fertility-sparing treatments are successfully used; however, these treatments can be offered only to a limited number of patients which meet the pathologic criteria for a conservative approach [119]. The indications for conservative treatment include the patient's desire to preserve fertility, no medical history of thrombosis, and no abnormal levels of hemostasis, a histologic diagnosis of grade 1 endometrioid adenocarcinoma by total endometrial curettage, no myometrial invasion or extrauterine spread of the disease observed by MRI, and hysteroscopy and total endometrial curettage must be repeated after 4–6 weeks of additional MPA therapy. Additionally, the expression of receptor for progesterone receptor (PR), PTEN gene, DNA mismatch repair gene MLH1 and phospho-AKT on tissue specimens may be useful for selecting patients fit for a conservative management [118, 120]. 

The opportunity of a demolitive surgery after delivery or after childbearing being no longer required is still a debated issue. Large multicenter trials are strongly warranted to better define the selection criteria for a conservative treatment, endocrine regimen of choice, the optimal dosing, the duration of treatment and follow-up protocols. Until now, the long-term outcome of children in utero exposed to oncological treatment modalities is poorly documented. Delivery should be postponed preferably until after a gestational age of 35 weeks. Further research including international registries for gynecologic carcinoma in pregnancy is urgently needed. The gathering of both available literature and personal experience suggested models for treatment of gynecologic carcinoma in pregnancy [121]. In any case, the patient should be accurately informed about the relatively high recurrence rates after complete response to hormone treatment and expectations for pregnancy.

## 13. Biomarker and Targeted Therapy

As previously stated, distinct molecular changes are associated with two subtypes, and these distinct molecular alterations also underscore prognostic differences. Therefore, active researches are enthusiastic about novel screening approaches that emerged from epigenetics, proteomics, and genomics in endometrial carcinoma. It is hopeful that the use of targeted therapies will improve the outcome for endometrial carcinoma.

 Nowadays, several novel tumor markers with increased sensitivity and specificity for endometrial carcinoma have been identified and are considered to help monitor response to therapy and to detect recurrent disease. These potential molecular biomarkers include HE4, CA125, Cyr61, p21, p53, Cathepsin-B, MMR, and ERR[alpha] progesterone receptor (PR)-A, which are estimated to contain potential value as prognostic factors for patients with endometrioid carcinoma [122–128]. Additionally, Bidus et al. reported two cell cycle checkpoint genes, CDC2, MAD2L1, and The ZIC2 zinc finger gene were associated with lymph node metastasis in endometrial carcinomas [129]. Currently, these tumor makers are utilized in this role with limited value. Further investigation in the role of biomarker for early detection of recurrent endometrial carcinoma and monitoring response to therapy is warranted. Gene expression profiling of the primary tumors in patients with endometrioid endometrial carcinomas seems promising for identifying genes associated with lymph node metastasis. Future studies should address whether the status of nodal metastasis can be determined from the expression profiles of preoperative tissue specimens.

With the progress of advanced gene techniques, it has become possible to identify potential molecular markers of endometrial carcinoma for its diagnosis, prognosis and therapy by global gene expression profiling. It may provide a foundation for the development of new diagnostic and prognostic markers and type-specific therapies against this common female genital malignant disease. Such procedure allowed us to give shape to preliminary gene expression profile typical for neoplastic tissue and to estimate protein expression of the most significant predictors of neoplastic transformation. Comparison of obtained data with tumor grade can reveal new markers of endometrial carcinoma useful in routine diagnostic procedures [23, 130]. 

Genes related to the endometrial carcinoma progression and metastasis can be identified by differential gene expression profile with cDNA microarray and high-risk endometrial carcinoma may be distinguished before surgery by hierarchical cluster analysis [131]. Similarly, the dysregulation of these miRNAs appeared to be involved in the progression of endometrial carcinoma [132]. Therefore, some researchers suggested that the cDNA and miRNAs microarray techniques may be feasible to generate gene expression profiles of endometrial carcinoma. Classification based on gene expression patterns may be more accurate than histological grade and FIGO stage classification in predicting the prognosis of tumors [133]. Further extended and functional studies of these new approaches are required to confirm the potential use of them in the endometrial carcinomas.

 With the applications of the target gene therapy, some valuable research had carried in advanced endometrial carcinoma. Since the year 2000, in advanced endometrial carcinoma, the GOG has conducted phase II trials with several molecular targeting agents including imatinib (Gleevec), trastuzumab (Herceptin), and gefitinib (Iressa) as single agents with negligible evidence of activity. The GOG does have active trials of chemotherapy with a molecular targeting agent such as bevacizumab (Avastin) in GOG 218, but there are no randomized molecular targeting agent trials in advanced endometrial carcinoma [134]. Some genes related with endometrial carcinoma prognosis have become a hopeful target for therapies in endometrial carcinoma, these targeting genes include mTOR inhibitors, EGFR tyrosine kinase inhibitors (erlotinib), and monoclonal antibodies to Her-2/neu (trastuzumab) [135–138]. However, acceptance of genetic consultation and testing is surprisingly low and deserves further investigation. For example, it is hypothesized that the HER-2/neu receptor could be used for targeted therapy in recurrent endometrial carcinoma. In a clinical trial, trastuzumab was of little clinical value in two cases of recurrent type II endometrial carcinoma based on the lack of response and changes in tumor biology [139]. In another trial, a multinomial design two-stage phase II study was performed to evaluate single-agent activity of erlotinib, an orally active, selective inhibitor of EGFR tyrosine kinase activity, in women who had advanced endometrial carcinoma with recurrent or metastatic disease, and were chemotherapy naive and received up to one line of prior hormonal therapy. The data showed that erlotinib is well tolerated with an overall objective response rate of 12.5% [136]. These reports underscore the importance of reassessment of targeted treatment in endometrial carcinoma. Yet, researchers still have a long way to go in order to reach the goal of applying the targeting gene therapy in clinical practice.

## 14. Prevention and Surveillance

In the follow-up of endometrial carcinoma patients, pain was the most common complaint in patients with recurrent disease, followed by vaginal bleeding, general malaise, loss of weight and intestinal complaints. With the evidence from randomized clinical trials we can conclude that a follow-up program in the first three years after primary treatment of endometrial carcinoma is helpful in detecting recurrent disease. 

In 2007, van Wijk et al., evaluated their clinical data of patients with recurrent endometrial carcinoma treated in the Erasmus Medical Centre in Rotterdam over a 20-year period [140]. He reported that patients with screen-detected recurrences had a 5-year survival rate of 62%. Patients with interval screening recurrences or recurrences detected by chance had a 5-year survival rate of 47%. Evaluating the patients with an endometrioid type of tumor separately, the 5-year survival rate for patients screen-detected recurrences is 68% and for patients with interval screening recurrences is 51% [140]. The reported median intervals to local and distant recurrent disease are consistent with those reported in the literatures [141, 142]. 

Tjalma et al. published an overview of 11 retrospective studies (evaluating 2866 patients) on routine follow-up of endometrial carcinoma. In these studies symptomatic recurrences ranged between 41% and 81% (mean 65%) of all recurrences [143]. Retrospective data from both Agboola group and Tjalma group suggest that there is no difference in survival between symptomatic and asymptomatic recurrences, or between women with recurrences detected during routine follow up visits and those with recurrences detected during the interval between routine visits [143, 144]. Furthermore, there is no economic or clinical justification for the routine use of the Pap smear and systematic radiography in the follow-up of patients with endometrial carcinoma [144, 145]. Centers advocating surveillance should focus on the detection of potentially curable vaginal recurrences, since isolated vaginal-vault recurrence of endometrial carcinoma is curable in up to 87% of cases, in patients previously not exposed to radiation [146]. 

 Tjalma et al. pointed out that because of a difference in survival between isolated vaginal recurrence and nonvaginal recurrences, 5-year survival, respectively 50% and 6%, it is important to identify isolated vaginal recurrences early. As the sensitivity of routine follow-up schemes appears very low, tailored follow-up protocols based on high risk and low risk for recurrence are suggested [143]. Low risk patients are generally defined as patients with adenocarcinoma IA grade 1 or 2 or IB grade 1, with a recurrence rate of just under 4%, whereas high risk patients have a recurrence rate of around 23% [147]. Salvesen et al. found a low risk group, with FIGO Stage IA/IB or patient age below 60 years at primary operation was identified in multivariate recurrence-free survival analysis. No asymptomatic recurrences were found in this group. Therefore, they concluded that low risk patients should be considered for less frequent follow-up [141]. However, van Wijk et al. reported of five low risk patients with recurrent disease, only one patient, suffering from distant recurrent disease, was symptomatic. Without a follow-up program for patients with low-risk endometrial carcinoma, recurrent disease would only have been detected after symptoms had developed in four of these five patients. It was discussed that there is no reason to use different follow-up scheme for these patients, despite our low number of patients with low risk disease. Improving patient education so that early symptoms of recurrence are reported appears eminently sensible, but may serve also to heighten anxiety amongst the majority who will never develop recurrent disease [142]. For patients who have evidence of metastatic disease at time of surgery, it is nowadays generally accepted that there is a survival benefit to be gained if all gross evidence of disease can be resected or at least debulked to leave small-volume residual disease [148].

 Most endometrial carcinomas are sporadic, but approximately 10% of cases have a hereditary basis [149–153]. Two genetic models have been suggested in the development of endometrial carcinoma: hereditary nonpolyposis colorectal carcinoma (HNPCC) syndrome, also known as Lynch II syndrome, and a predisposition for endometrial carcinoma alone. Both are autosomal dominant inherited carcinoma susceptibility syndrome caused by a germline mutation in one of the deoxyribonucleic acid (DNA) mismatch repair genes [153]. It is associated with early onset of carcinoma (age younger than 50 years) and the development of multiple carcinoma types, particularly colon and endometrial carcinoma. Women with Lynch syndrome have a 40-60% risk of endometrial carcinoma, which equals or exceeds their risk of colorectal carcinoma. In addition, they have a 12% risk of ovarian carcinoma. Despite limited information on the efficacy of surveillance in reducing endometrial and ovarian carcinoma risk in women with Lynch syndrome, the current gynecologic carcinoma screening guidelines include annual endometrial sampling and transvaginal ultrasonography beginning at age 30–35 years [154]. But the cost effectiveness of this screening has not been proven either. An alternative approach is primary prevention by using a progestogen device in utero, such as the Mirena IUCD. This merits full evaluation [155].

In addition, risk-reducing surgery consisting of prophylactic hysterectomy and bilateral salpingooophorectomy should be offered to women aged 35 years or older who do not wish to preserve their fertility [154]. Schmeler et al. reported a retrospective analysis of women with known germline mutations associated with Lynch syndrome. Sixty-one participants underwent prophylactic hysterectomy and were compared to over 200 matched controls with similar mutations that did not have preventive surgery. Endometrial carcinoma was eventually diagnosed in 33% of the controls with no cases in the prophylactic surgery group [156]. Pistorius et al. report detected asymptomatic muscle invasive endometrial carcinoma in two of four women who underwent prophylactic hysterectomy after requiring surgery for Lynch syndrome related colorectal carcinoma [157]. In 2006 a multiinstitutional, matched case-control study found that prophylactic hysterectomy with bilateral salpingo-oophorectomy is an effective primary preventive strategy in women with HNPCC syndrome [156]. Based on these observations, surgery as primary prevention for women at high risk due to known germline lesions or history of Lynch syndrome related malignancies may yield a meaningful reduction in progression to endometrial carcinoma.

## 15. Summary

Endometrial carcinoma is a low-grade curable malignancy and most patients who present with early disease have excellent survival rate. Endometrial carcinoma remains a management challenge, presenting with a full spectrum of disease ranging from that with excellent prognosis and high curability to aggressive disease with poor outcome. There are many debates and controversies about optimal treatment for women with different staging endometrial carcinoma. How do we summarize the current recommendations and how do we proceed? Clinicians must balance delivering adequate therapy while attempting to minimize treatment morbidity and must always be weighed carefully. 

 Improved understanding of the mechanisms of carcinogenesis may help identify molecular signatures that could predict biologic behavior of individual disease presentations and discover potential molecular candidates for targeted therapies. Total hysterectomy and bilateral salphingo-oophorectomy is the primary operative procedure. Pelvic lymhadenectomy is performed in most centers on therapeutic and prognostic grounds and to individualize adjuvant treatment. Women with endometrial carcinoma can be readily segregated intraoperatively into “low-risk” and “high-risk” groups to better identify those women who will most likely benefit from thorough lymphadenectomy. Postoperative irradiation is used to reduce pelvic and vaginal recurrences in high risk cases. Treatment planning should be conservative in order to reduce patients' morbidity and overtreatment while maintaining acceptable recurrence and survival rates. Progression in diagnostic imaging, radiation delivery systems, and systemic therapies potentially can improve outcomes while minimizing morbidity. The availability of new hormonal and biological agents presents new opportunities for therapy. Novel strategies for screening and prevention also hold promise for reducing incidence and mortality of this disease. The current evidence suggests that there remain avenues to improve management and we need to continue rigorous investigation to identify and implement the best available practice. Research in the next ten years should provide valuable new strategies not only for treatment but also for prevention.

## Figures and Tables

**Table 1:** WHO histological classification of endometrial carcinoma.

*Endometrioid adenocarcinoma*
Variants: with squamous differentiation
Villoglandular
Secretory
with ciliated cells

*Other adenocarcinomas*
Mucinous carcinoma
Serous carcinoma
Clear-cell carcinoma
Mixed carcinoma
Squamous-cell carcinoma
Transitional-cell carcinoma
Small-cell carcinoma
Undifferentiated carcinoma
